# B. T. Mason, D.D.S.

**Published:** 1890-11

**Authors:** 


### Obituary.

### B. T. MASON, D.D.S.

THE death of Dr. B. T. Mason, of Phœnix, N. Y., which took place September 3, leaves a vacancy in the profession of the Fifth District, New York, not readily filled.

Dr. Mason was a member of the State Society, and also of the Fifth District Society, and was twice made president of the latter organization. He graduated at the Department of Dentistry, University of Pennsylvania, in 1881, and settled in Phœnix, where he acquired a large practice. He was active outside of his profession, having been secretary of the Phœnix Union Agricultural Society, and was also a member of the Board of Education.

His funeral took place at Gilbert's Mills, and was largely attended by the Masonic order, of which he was a prominent member.

